# A prompt technique to identify fluoroquinolone antibiotics (FQs) residue in the environments

**DOI:** 10.1016/j.mex.2025.103287

**Published:** 2025-03-26

**Authors:** Nahin Mostofa Niloy, Fahmida Parvin, Shafi M Tareq

**Affiliations:** a Hydrobiogeochemistry and Pollution Control Laboratory, Department of Environmental Sciences, Jahangirnagar University, Dhaka 1342, Bangladesh; b Centre for Coastal Biogeochemistry, School of Environment, Science and Engineering, Southern Cross University, Lismore, New South Wales, Australia

**Keywords:** Fluoroquinolone antibiotics, Fluorescence spectroscopy, Humic substance, Excitation-emission Matrix (EEM), PARAFAC model, Distinct identification of FQs antibiotics and ubiquitous humic substance in the mixture

## Abstract

The widely used fluoroquinolone (FQ) antibiotics have become emerging contaminants due to their unconstrained usage. A prompt, straightforward, précised, and advanced technique named fluorescence spectroscopy was applied to characterize and semi-quantify FQs. However, excitation wavelengths on the comparable ranges of FQs and ubiquitous humic substance (HS) complicate their identification in the mixture. This study develops a simple spectral technique that affirms the individual identification of FQs and HS in the natural environment. The following characteristics and methods can demonstrate the traces of FQs in the environment-

• Presence of fluorophore at Excitation/Emission = 225–230/285–295 nm wavelength in excitation-emission matrix (EEM) and parallel factor analysis (PARAFAC) models.
• FQs and HS have fluorophores at excitation wavelengths of 275 nm and 325 nm in PARAFAC analysis. Unlike HS, the notable spectral presence in <350 nm emission during 275 nm excitation in FQs ensures their distinct identification in the mixture.
• FQ affirming component intensity at Excitation/Emission = 225–230/285–295 nm and the intensity <350 nm emission during 275 nm excitation are considered in conjunction to quantify the amount of least FQ in any environmental sample.

## Graphical abstract

**Figure:**
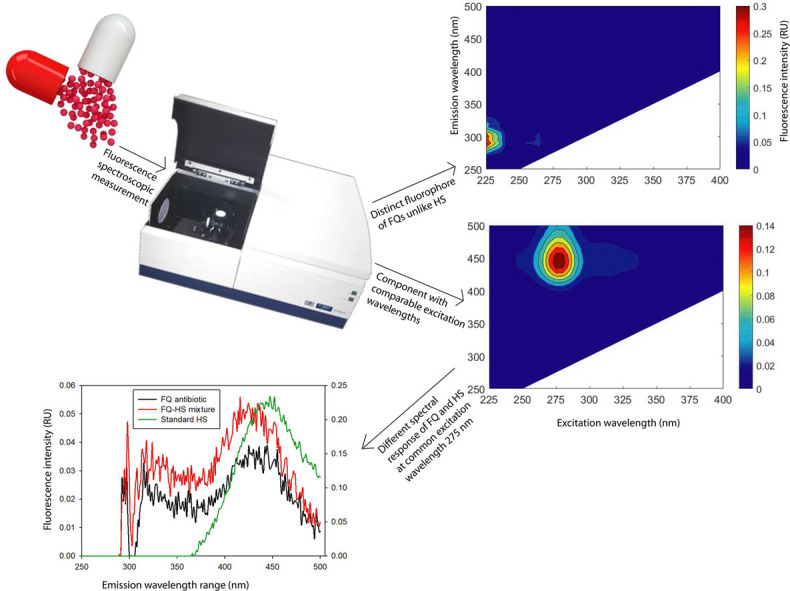

Specifications table

**Table:** 

Subject Area:	Environmental chemistry
More specific subject area:	Emerging contaminants
Method name:	Distinct identification of FQs antibiotics and ubiquitous humic substance in the mixture
Name and reference of original method:	N.M. Niloy, F. Parvin, S.M. Tareq, Spectral characterization, degradation behavior, quenching, and semi-quantification of fluoroquinolone antibiotics in the antibiotic-humic mixture using fluorescence spectroscopy, Science of the Total Environment 935 (2024). https://doi.org/10.1016/j.scitotenv.2024.173346.
Resource availability:	N/A

## Background

The wide applications of pharmaceutical compounds in human and veterinary treatments have become emerging threats to human health and the ecosystem because of their intensifying detection in the environment [[1], [2], [3]]. The continual dispensing of such contaminants can further increase microbial resistance [4]. Fluoroquinolones (FQ) are wide-spectrum antibiotics with substantial oral bioavailability and unconscionable application in hospitals and households [5]. A 5.2 % annual growth ensures the third-largest contribution by FQs in the global antibiotics market [6]. It is also anticipated that the revenue from the FQs market will expand to $10.1 billion from $6.8 billion by 2027 [6]. However, 70 % of the FQs antibiotics are discharged into the environment as unmetabolized [2]. The high adsorption affinity and pseudo-persistent character of FQs were found to promote microbial resistance and affect terrestrial and aquatic species [2]. Such widespread distributions of unmetabolized FQs demand advanced, prompt, simple, and convenient techniques for their characterization and quantification in the environment.

Prolonged sample pre-treatment and application of different chemicals in liquid chromatography and mass spectroscopy have turned such measurement techniques time-consuming and complex [7,8]. However, an advanced and multivariate three-dimensional technique named fluorescence spectroscopy has conquered the drawbacks of liquid chromatography and mass spectrometry [9,10]. Prompt, sensitive, and powerful fluorescence spectroscopy hardly requires any sample pre-treatment for measurement [9,10]. Fluorescence spectroscopy portrays a three-dimensional landscape with the sample's dissolved organic components (DOM), including excitation/emission wavelength ranges and concentrations. Parallel factor analysis (PARAFAC), another advanced and multivariate analytical approach, efficiently separates individual DOM components from the EEM model according to the distinct excitation/emission wavelength position. The characterization and identification of DOM components are précised and validated in EEM-PARAFAC and, therefore, was considered an utterly fit technique in this study.

## Method details

### Sample preparation and experimental plan

Ciprofloxacin, Levofloxacin, and Moxifloxacin, three broadly used FQ antibiotics, were considered to study and were labelled A1, A2, and A3, respectively. Standard humic substance (HS) obtained from the International Humic Substance Society (IHSS) was also considered for this study.

A stock solution of 1 mg/L was prepared for each FQ antibiotic and HS. Each FQ stock was mixed with HS stock and labelled A1-H, A2-H, and A3-H. Individual and mixed stock solutions of FQ and HS were studied for photo-irradiation and dark experiments for 10 days, of which samples were collected for fluorescence measurement on Day 1, Day 2, Day 3, Day 4, Day 7, and Day 10. Stock solutions were placed in mouth-sealed glass vials and kept under sunlight during the photo-irradiation experiment. This study was performed in October and November, while mean solar radiation was 181.3 Cal/cm^2^/min and 180.03 Cal/cm^2^/min, and mean sunshine for 5.9 and 6.9 hours in Dhaka [11]. For the dark experiment, glass vials were wrapped in pre-combusted foils and placed in a dark and tidy place. The mean temperature during photo-irradiation and dark experiments were 33–34°C and 22–23°C, respectively.

### Sample measurement in fluorescence spectrophotometer

Samples were measured for fluorescence properties using a Hitachi F-4600 fluorescence spectrophotometer. Excitation (Ex) and emission (Em) wavelength ranges were set at 225–400 nm and 250–500 nm with wavelength intervals at 5 nm and 1 nm, respectively, during sample scanning in a fluorescence spectrophotometer. The scanning speed was 1200 nm/min, and the photomultiplier tube voltage and silt range of excitation and emission band were set at 700 V and 5 nm, respectively, during measurement. Instrumental response during sample scanning was set as auto. Ultrapure Milli-Q water was used as a blank in the study. The acquired sample data from the fluorescence spectrophotometer were at an arbitrary unit, which was further converted to a Raman unit (RU) using the Raman peak position of blank Milli-Q water.

### Three-dimensional EEM and PARAFAC model with the fluorescence dataset

EEM and PARAFAC models were performed in MATLAB software with the sample data found in the fluorescence spectrophotometer. N-Way package containing DOMFluor (v1.7) toolbox was installed in MATLAB during PARAFAC analysis. Before EEM and PARAFAC analyses, blank Milli-Q was subtracted from sample data to remove Raman and Rayleigh scattering [12]. Outliertest was performed to remove samples with noisy wavelengths [12]. Split Half analysis was conducted to validate the dataset for PARAFAC analysis and identify the exact number of fluorescent components [12]. The intensities of identified components were read at RU [12].

### Characterization and identification of FQs and HS in the mixture

**Fig. 1:**
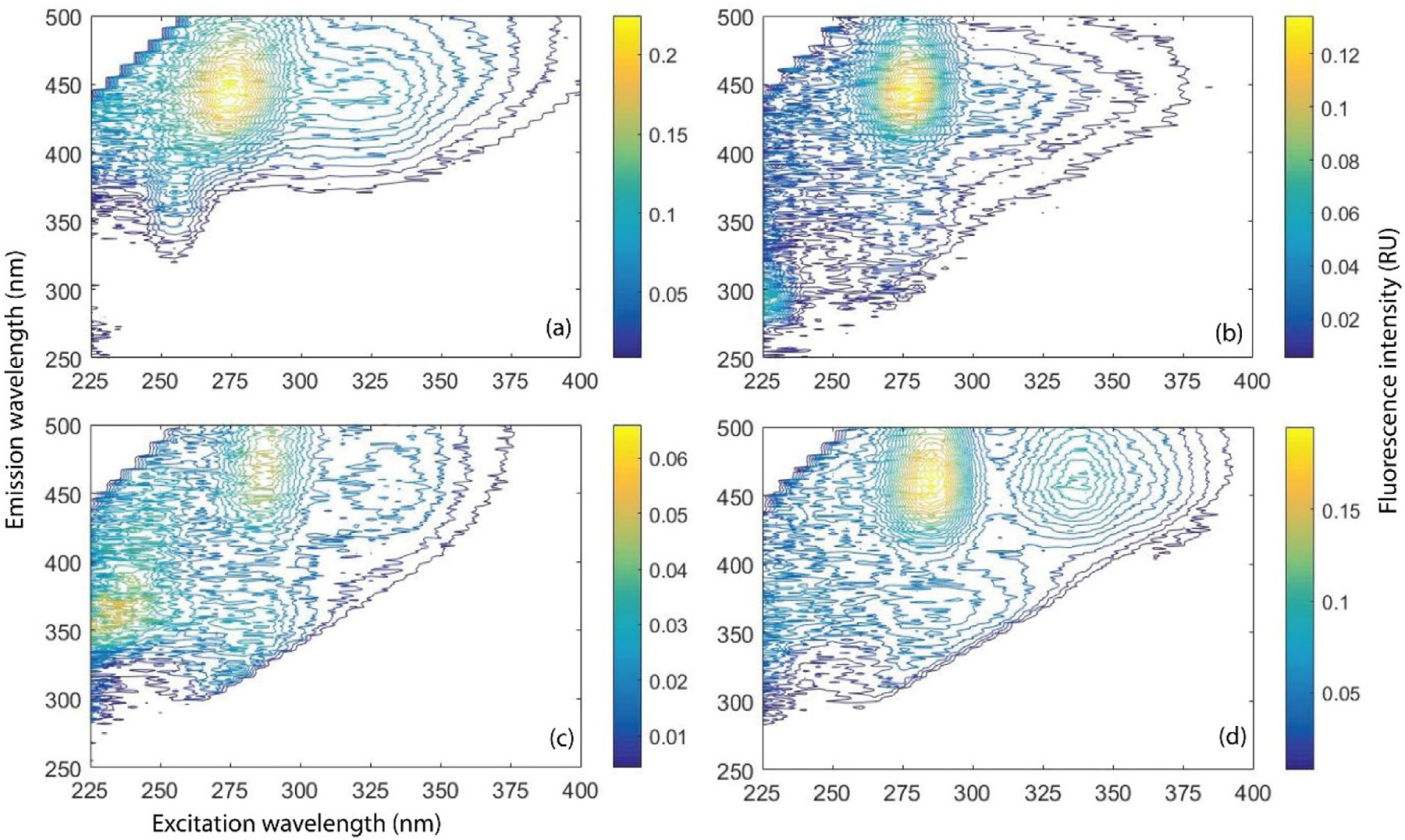
Fluorophores in (a) HS (Humic standard), (b) A1 (Ciprofloxacin), (c) A2 (Levofloxacin), and (d) A3 (Moxifloxacin) during photo-irradiation and dark experiments identified by EEM model.

**Fig. 2:**
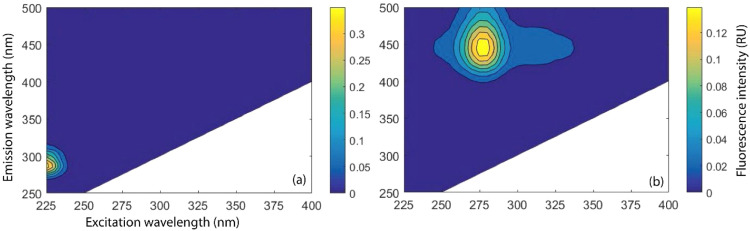
Fluorescent components in FQ antibiotics and HS identified by PARAFAC analysis.

EEM model identified fluorophores at Ex/Em = 275/446 nm and 325/439 nm in HS and FQ antibiotics during photo-irradiation and dark experiments (Fig. 1). In PARAFAC analysis, a DOM component with Ex/Em = 225–230/285–295 nm wavelength was identified in FQ antibiotics, which was utterly absent in HS (Fig. 2a). The presence of such distinct DOM component in FQs firmly ensured separate identification of FQ and HS in the mixture (Fig. 2a). However, a commonly identified DOM component having fluorophores at excitation wavelength 275 nm and 325 nm in FQ and HS complicate their distinct separation (Fig. 2b).

**Fig. 3:**
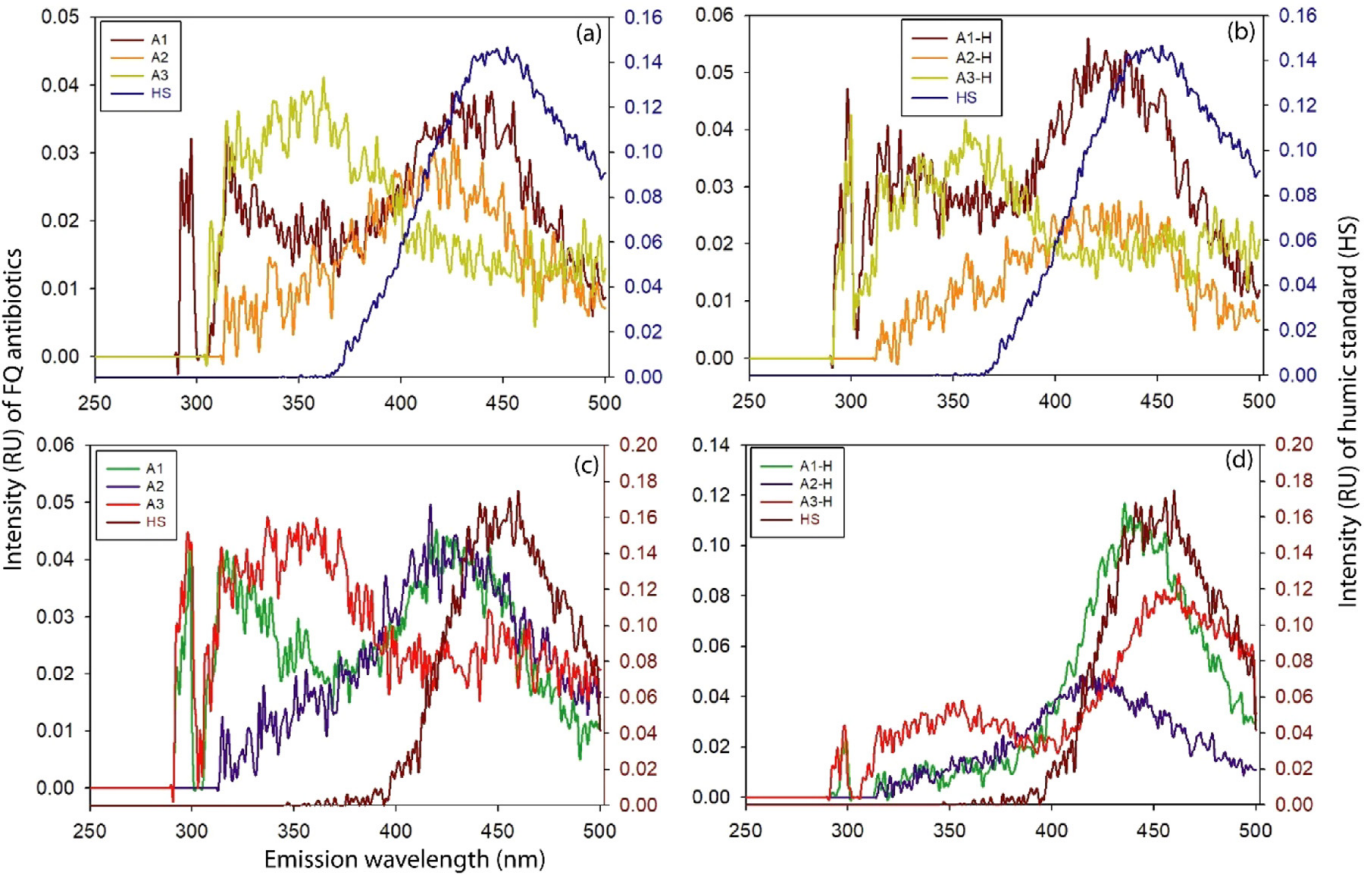
Spectral response through the entire emission range during 275 nm excitation in HS and FQ antibiotics: (a) FQs and HS in photo-irradiation experiment (b) FQ-HS mixture in photo-irradiation experiment (c) FQs and HS in dark experiment (d) FQ-HS mixture in dark experiment.

To resolve ambiguity in separation and identify spectral differences at typical excitations 275 nm and 325 nm, entire emission (250–500 nm) at such two excitation wavelengths were plotted for both HS and FQs (Fig. 3). In such plotting, FQs showed notably different spectral response than HS at 275 nm excitation wavelength (Fig. 3). HS hardly had any spectrum at short emission (<350 nm) wavelength during both 275 nm and 325 nm excitations (Fig. 3). On the contrary, unlike to 325 nm excitation, FQs showed uninterrupted spectrum at short emission (<300–350 nm) wavelength during 275 nm excitation both in photo-irradiation and dark experiments (Fig. 3). The spectral presence at short emission (<300–350 nm) wavelength during 275 nm excitation in FQs ensured their distinct identification in the HS containing mixture.

### Semi-quantification of FQ antibiotics in FQ-HS mixture

**Fig. 4:**
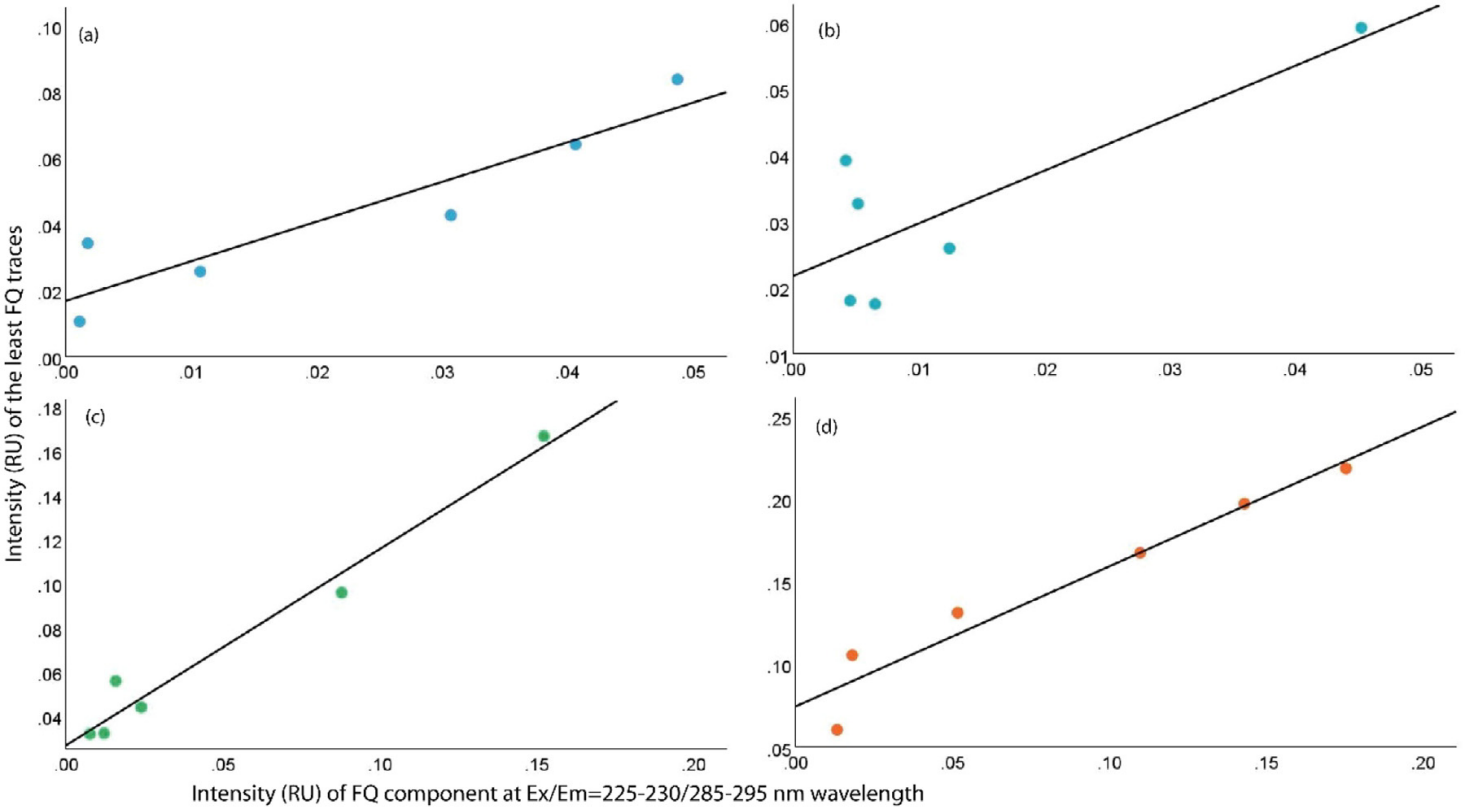
Correlation between component Ex/Em = 225–230/285–295 nm and least FQ traces: (a) A1 (Ciprofloxacin), (b) A2 (Levofloxacin), (c) A3 (Moxifloxacin) and (d) studied FQs altogether.

Fluorescence intensity at <350 nm emission during 275 nm excitation wavelength and component intensity at Ex/Em = 225–230/285–295 nm wavelength were summed to ensure the least abundance of FQs in the sample. Such integrated intensity was plotted against the component intensity at Ex/Em = 225–230/285–295 nm wavelength to identify the strength of their correlation (Fig. 4). Such correlation was found linear, significant, and strong for all three FQ antibiotics individually and altogether affirming the sheer possibility to identify least FQ intensity in any sample using such semi-quantification technique (Table 1). The obtained equation from such significant correlations can efficiently identify the least intensity of a particular FQ or FQs from the corresponding component intensity at Ex/Em = 225–230/285–295 nm wavelength (Table 1).

**Table 1:** Correlation equation between component intensity at Ex/Em = 225–230/285–295 nm (x axis) and least intensity of FQ traces (y axis). Least intensity of FQ is the sum of component intensity at 225–230/285–295 nm and intensity <350 nm emission during 275 nm excitation.

Sample	Pearson correlation, r	Significance, p	Correlation equation
A1	0.93	<0.05*	y=1.1977x+0.0168
A2	0.81	<0.05*	y=0.7934x+0.0218
A3	0.99	<0.05*	y=0.8892x+0.0267
Studied FQs	0.97	<0.05*	y=0.8461x + 0.0746

⁎ Correlation is significant at the <0.05 level.

### Method validation

The distinct FQs identification technique was applied to the different lake water samples. The studied four lakes were labelled L1, L2, L3 and L4. L1 and L2 were located near a hospital and a residential area. L3 was a reservoir lake, and L4 was the point source of a natural stream.

**Fig. 5:**
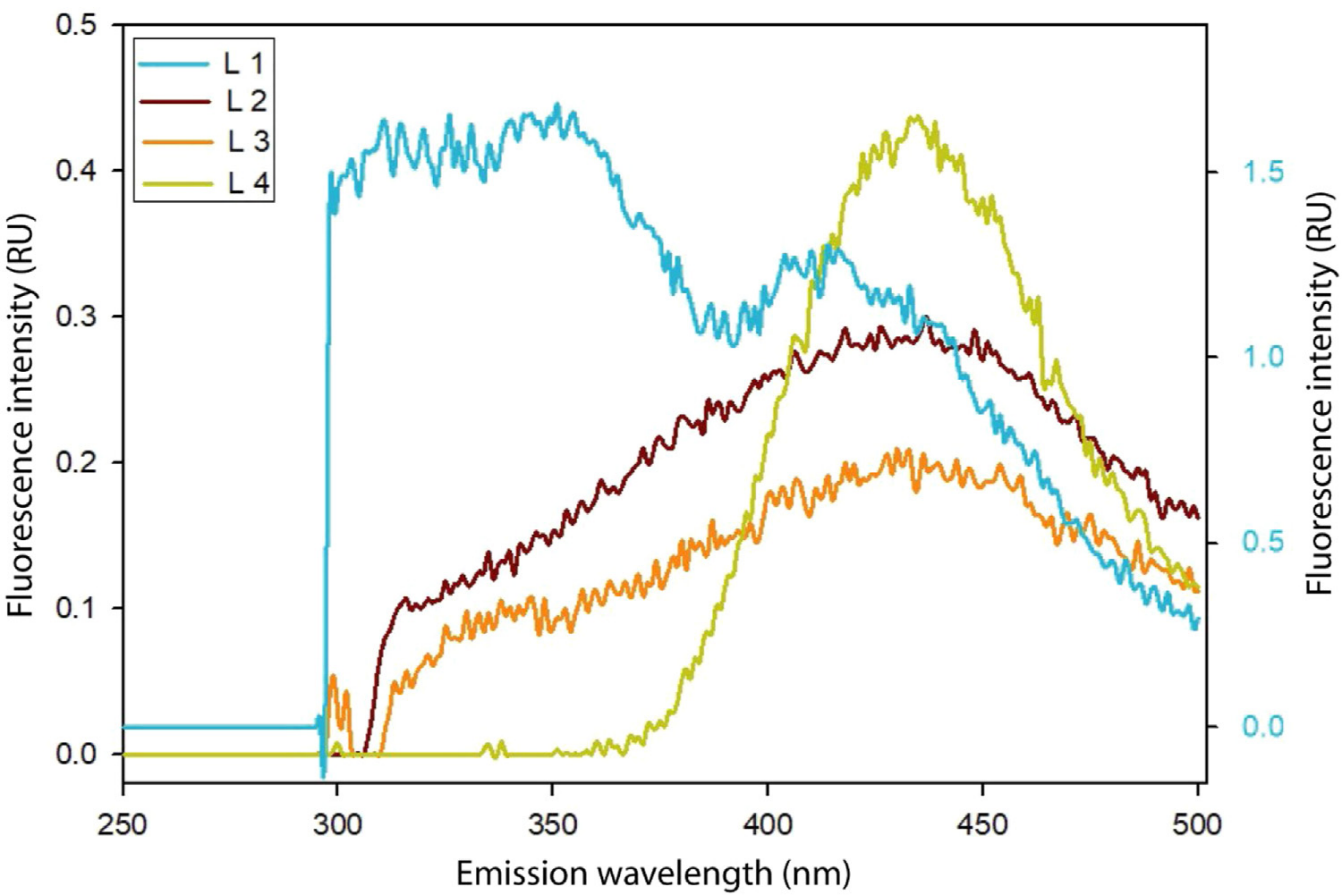
Spectral pattern through the entire emission range during 275 nm excitation in lake water samples. L1 was located near a hospital, and L2 was inside a residential area. L3 and L4 were a reservoir lake and a stream, respectively.

EEM identified fluorophores at Ex/Em = 230/290 nm in L1, Ex/Em = 265/431 nm and 315/405 nm in L2, Ex/Em = 260/434 nm and 315/408 nm in L3, and Ex/Em = 275/434 nm and 320/431 nm in L4 (Table 2). Among the identified components in lake water by the PARAFAC model, two components had fluorophores at Ex/Em = 230/292 nm and Ex/Em = (275/434 nm, 320/435 nm) (Table 2). FQ-affirming component (Ex/Em = 230/292 nm) was absent in L4, ensuring such lake water is FQ antibiotic-free (Table 2). Moreover, water in L4 hardly had any spectra at the short emission range (<350 nm) during 275 nm excitation, affirming that component Ex/Em = (275/434 nm, 320/435 nm) in this lake was from humic substance, not from FQs (Fig. 5). On the contrary, spectral presence at <350 nm emission during excitation wavelength 275 nm in L1, L2, and L3 affirmed the FQs existence in such lake waters (Fig. 5).

**Table 2:** Fluorophores and components identified by EEM and PARAFAC model analyses in lake water samples.

Lake Samples	Wavelength of fluorophores (Ex/Em) identified in EEM model	Components identified in PARAFAC analysis	
		Component wavelength (Ex/Em)	Component intensity (RU)
L1	230/290 nm	230/292 nm	4.6 RU
L2	265/431 nm, 315/405 nm	230/292 nm	0.03 RU
		275/434 nm, 320/435 nm	0.04 RU
L3	260/434 nm, 315/408 nm	230/292 nm	0.06 RU
		275/434 nm, 320/435 nm	0.07 RU
L4	275/434 nm, 320/431 nm	275/434 nm, 320/435 nm	0.4 RU

Spectral intensity <350 nm emission at 275 nm excitation and intensity of component Ex/Em = 230/292 nm were summed to identify the least FQ presence in L1, L2 and L3. Least FQ intensity in such three lakes followed the descending order: L1 (6.2 RU) > L2 (0.18 RU) > L3 (0.16 RU). The FQ intensity affirmed its presence in large amounts at the nearby medical lake water compared to the residential area and reservoir lake.

## Conclusion

Fluorophore at Excitation/Emission = 225–230/285–295 nm wavelength and spectral presence at short emission wavelength <350 nm at 275 nm excitation have specified the characterization, identification, and semi-quantification of FQs in the environment. Because of its prompt, simple, and précised methodology, such identified techniques in fluorescence spectroscopy can efficiently contribute to any control and action plans to manage FQs in the environment. Considering the widespread rapid distribution of unmetabolized FQs in the environment, this technique is badly required nowadays.

## Limitations

Not applicable

## Ethics statements

Not applicable

## CRediT authorship contribution statement

**Nahin Mostofa Niloy:** Methodology, Investigation, Data curation, Writing – original draft. **Fahmida Parvin:** Methodology, Investigation, Writing – review & editing. **Shafi M Tareq:** Conceptualization, Methodology, Software, Validation, Data curation, Supervision, Writing – review & editing.

## Declaration of competing interest

The authors declare that they have no known competing financial interests or personal relationships that could have appeared to influence the work reported in this paper.

## Data Availability

Data will be made available on request.

## References

[1] Kulikova N.A., Solovyova A.A., Perminova I.V. (2022). Interaction of antibiotics and humic substances: environmental consequences and remediation prospects. Molecules..

[2] Van Doorslaer X., Dewulf J., Van Langenhove H., Demeestere K. (2014). Fluoroquinolone antibiotics: an emerging class of environmental micropollutants. Sci. Total Environ..

[3] Shammi M., Rahman M.M., Ali M.L., Khan A.S.M., Siddique M.A.B., Ashadudzaman M., Bodrud-Doza M., Alam G.M.M., Tareq S.M. (2022). Application of short and rapid strategic environmental assessment (SEA) for biomedical waste management in Bangladesh. Case Studies Chem. Environ. Eng..

[4] Allen H.K., Donato J., Wang H.H., Cloud-Hansen K.A., Davies J., Handelsman J. (2010). Call of the wild: antibiotic resistance genes in natural environments. Nat. Rev. Microbiol..

[5] Michalak K., Sobolewska-Włodarczyk A., Włodarczyk M., Sobolewska J., Woźniak P., Sobolewski B. (2017). Treatment of the fluoroquinolone-associated disability: the pathobiochemical implications. Oxid. Med. Cell Longev..

[6] Researchdive, Antibiotics Market Report, (2021).

[7] Seifrtová M., Aufartová J., Vytlačilová J., Pena A., Solich P., Nováková L. (2010). Determination of fluoroquinolone antibiotics in wastewater using ultra high-performance liquid chromatography with mass spectrometry and fluorescence detection. J. Sep. Sci..

[8] Hossain A., Nakamichi S., Habibullah-Al-Mamun M., Tani K., Masunaga S., Matsuda H. (2018). Occurrence and ecological risk of pharmaceuticals in river surface water of Bangladesh. Environ. Res..

[9] Hudson N., Baker A., Reynolds D. (2007). Fluorescence analysis of dissolved organic matter in natural, waste and polluted waters-a review. River. Res. Appl..

[10] Stedmon C.A., Markager S. (2005). Resolving the variability in dissolved organic matter fluorescence in a temperate estuary and its catchment using PARAFAC analysis. Limnol. Oceanogr..

[11] Khan S.H., Towfiq-ur-Rahman S.Hossain (2012). A brief study of the prospect of solar energy in generation of electricity in Bangladesh. Cyber Journals: Multidiscip. J. Sci. Technol. J. Select. Areas Renew. Sustain. Energy (JRSE).

[12] Stedmon C.A., Bro R. (2008). Characterizing dissolved organic matter fluorescence with parallel factor analysis: A tutorial. Limnol. Oceanogr. Methods.

